# Supplementary material for: Social disparities in unplanned 30-day readmission rates after hospital discharge in patients with chronic health conditions: A retrospective cohort study using patient level hospital administrative data linked to the population census in Switzerland
Source: PLoS One. 2022 Sep 22;17(9):e0273342. doi: 10.1371/journal.pone.0273342 (PMC9499293; doi:10.1371/journal.pone.0273342)
Supplement: S1 File — (PDF) [file pone.0273342.s001.pdf]

S1_File

Auszug Variablen-Liste, BFS-Daten

Variables of the SIHOS-Database used for the paper *Social disparities in unplanned 30-day readmission rates after hospital discharge in patients with chronic health conditions: A retrospective cohort study using patient level hospital administrative data linked to the population census in Switzerland (2022)*

Benötigte Daten

BFS-Daten (falls im Antrag vorgesehen)

1. *Benötigte Variablen (diese sind vollständig aufzulisten, wobei sich die Schreibweise nach dem entsprechenden Datenkatalog richten muss)*
2. *Referenzperiode*
3. *Verknüpfungsidentifikator*

Es müssen Variablen aus den folgenden Datenquellen verknüpft werden:
- STATPOP
- Strukturerhebung
- Medizinische Statistik der Krankenhäuser (MS)
- MS: Verknüpfung über die Jahre (Mehrfachaufenthalte)

1) Benötigte Variablen

**STATPOP-Nr**	**Bezeichnung**	**Kategorien/Bemerkung**
	anonymisierter individueller Identifikator	
Abgeleitet aus 31	Alter	in Jahren
33	Geschlecht	1 weiblich<br>2 männlich
412	Staatsangehörigkeit	1 Schweiz<br>2 EU/EFTA<br>3 Anderer europäischer Staat<br>4 Aussereuropäischer Staat<br>5 Unbekannt
624	Haushaltsart	1 Privathaushalt
**Struktur-erhebung-Nr.**	**Bezeichnung**	**Kategorien/Bemerkung**
	anonymisierter individueller Identifikator	
1	1. Hauptsprache (sprache_1)<br>2. Hauptsprache (sprache_2)<br>3. Hauptsprache (sprache_3)	1 Deutsch<br>2 Französisch<br>3 Italienisch<br>4 Englisch<br>5 Andere Sprachen
6 und 7	Migrationshintergrund	1 Schweizer/-in ohne Migrationshintergrund<br>2 Schweizer-/in mit Migrationshintergrund<br>3 Ausländer/-in der ersten Generation<br>4 Ausländer/-in der zweiten und höheren Generation<br>5 Migrationshintergrund unbekannt
8	höchste abgeschlossene Ausbildung	1 Sekundarstufe 1<br>2 Sekundarstufe 2<br>3 Tertiärstufe
Abgeleitet	Haushaltstyp	1 Einpersonenhaushalte<br>2 Nichtfamilienhaushalte mit mehreren Personen<br>3 Paare ohne Kinder im Haushalt<br>4 Paare mit Kindern im Haushalt<br>5 Elternteile mit Kindern im Haushalt<br>6 Mehrfamilienhaushalte
**MS Variablen Nr.**	**Bezeichnung**	**Bemerkung**
**Einschlusskriterien:**<br>Teilnahme des Patienten/der Patientin an einer der Strukturerhebungen 2010-2014 UND 1.3.V01 (Behandlungsart) = 3 (stationär) UND (Statistikfall = A oder Statistikfall=B) , mit allen Wiedereintritten		
**Minimaldatensatz (MB-Datensatz)**		

0.2.V01a	anonymisierter individueller Identifikator	
ID	Anonyme Fallnummer	
0.0.V01	Jahr	Um zeitliche Trends in den Datenanalysen zu berücksichtigen
EintrittErhebungsjahr	Eintritt erfolgte im Erhebungsjahr MS	Für analysenspezifische Selektion der Fälle
Austritt Erhebungsjahr	Austritt erfolgte im Erhebungsjahr MS	Für analysenspezifische Selektion der Fälle
**0.1.V02a**	Anonyme Bertriebsnummer	Damit berücksichtigt werden kann, dass Hospitalisationen im gleichen Spital einen Cluster bilden (z.B. in Random-Effects-Modellen)
**0.2.V02**	Kennzeichnung des Statistikfalls	A, B, C; für Selektion Fälle
0.3.V03	Patientengruppen-Datensatz	
1.1.V01	Geschlecht	Für Plausibilisierung mit STATPOP
1.1.V03	Alter bei Eintritt in Jahren	Für Plausibilisierung mit STATPOP
0.0.V12	Schweizer - Nicht-Schweizer	Für Plausibilisierung mit STATPOP
1.3.V01	Behandlungsart	Für Selektion Fälle
1.3.V02	Klasse	
1.4.V01	Hauptkostenstelle	
1.5.V03	Aufenthalt nach Austritt	
0.0.V05-2	Aufenthaltsdauer (SwissDRG)	
**Patientengruppen-Zusatzdaten (MD-Datensatz)**		
	ID Anonyme Fallnummer	
4.2.V010	MD-Hauptdiagnose	ICD-10-GM-Kode
4.2.V020	MD-Zusatz zu Hauptdiagnose	ICD-10-GM-Kode
4.2.V030<br>4.2.V040<br>4.2.V…	MD-1.Nebendiagnose<br>MD-2. Nebendiagnose<br>etc. für alle Nebendiagnosen	ICD-10-GM-Kode
**Sequenzvariablen**		
InputFile	Herkunftsfile des Records	0=MS;
s_ms	Sequenznummer der MS-Fälle (abgeleitet von 1.2.V01/ 1.5.V0)	Nr. der Behandlungssequenz unter Berücksichtigung der MD-Kennzeichnung von Wiedereintritten (Zwischenaustritte 4.7.V01, 4.7.V11 etc. und Wiedereintritte 4.7.V.02, 4.7.V12 etc.), inkl. Grund der Wiedereintritte (4.7.V03, 4.7.V13 etc.)

2. Referenzperioden:

- STATPOP/Strukturerhebung: 2010 – 2014
- Medizinische Statistik: 2010 − 2016

3. Verknüpfungsidentifikator für die Verknüpfung

- von Strukturerhebung und STATPOP: AHVN13
- von Strukturerhebung / STATPOP mit MS: anonymer Verbildungscode, der für die STATPOP und Strukturerhebung via Stichprobenregister gebildet werden kann.

Bedingungen:

Die Daten werden ausschliesslich für den im Vertrag definierten Zweck verwendet und nicht an Dritte weitergegeben. Die Daten werden nach Abschluss des Projekts gelöscht.

Die Daten werden an einem gesicherten Ort gespeichert, der ausschliesslich den Projektmitarbeitenden Zugang erlaubt.
