# Supplementary material for: Social disparities in unplanned 30-day readmission rates after hospital discharge in patients with chronic health conditions: A retrospective cohort study using patient level hospital administrative data linked to the population census in Switzerland
Source: PLoS One. 2022 Sep 22;17(9):e0273342. doi: 10.1371/journal.pone.0273342 (PMC9499293; doi:10.1371/journal.pone.0273342)
Supplement: S1 Table — (PDF) [file pone.0273342.s002.pdf]

S1 Table. Odds ratios of fully adjusted logistic regression for risk of unplanned 30-day readmission (all-conditions model C) with interaction chronic condition*education level (N=62,109)

Outcome: risk for 30-day readmission	Model C with CHC*education level			
	Sig.	OR	95% CI Lower	Upper
Education level				
tertiary (ref.)	0.252			
upper secondary	0.194	1.3	0.875	1.933
compulsory	0.097	1.397	0.941	2.072
Insurance class				
mandatory (ref.)				
(Semi-)private	0.035	0.893	0.804	0.992
Household type				
Living with others (ref.)				
Living alone	0.149	1.077	0.974	1.192
Sex				
Men (ref.)				
Women	0.031	0.891	0.803	0.989
Language skills				
At least regional language or English (ref.)				
Not regional language and no English	0.41	1.06	0.923	1.217
Age				
<= 55 years	<.001			
56-65 years	0.001	1.318	1.116	1.556
67-74 years	<.001	1.6	1.357	1.886
75+ years	<.001	1.98	1.686	2.326
Chronic health condition				
Ischaemic heart disease (ref.)	<.001			
Lung cancer	0.243	1.424	0.787	2.575
Colon cancer	0.061	0.471	0.214	1.037
Breast cancer	0.007	0.42	0.223	0.791
Prostate cancer	0.989	0.997	0.627	1.584
Diabetes with/without complications	0.91	0.964	0.512	1.816
Acute myocardial infarction	0.01	0.503	0.298	0.849
Acute cerebrovascular diseases	0.065	0.593	0.34	1.032
Congestive heart failure	<.001	0.395	0.244	0.642
COPD or asthma	0.067	0.494	0.232	1.051
Osteoarthritis	<.001	0.294	0.188	0.461
Back problems and disc order	<.001	0.336	0.21	0.537
Comorbidity				
NSD, centered by CHC	<.001	1.145	1.108	1.183
Mental comorbidity: no (ref.)				
Mental comorbidity: yes	0.02	1.188	1.028	1.373
Previous hospital stay last 6 months				
No (ref.)				
Yes	<.001	1.771	1.587	1.976
Length of hospital stay (LOS)				
LOS, centred by CHC, Q1 (ref.)	<.001			
LOS, centred by CHC, Q2	0.412	0.943	0.82	1.084
LOS, centred by CHC, Q3	0.257	1.079	0.946	1.232
LOS, centred by CHC, Q4	<.001	1.637	1.445	1.856
CHC*Education level				
Ischaemic heart disease (ref.)	0.074			
Lung cancer*upper secondary	0.213	1.516	0.788	2.919
Lung cancer*compulsory	0.291	1.429	0.737	2.769
Colon cancer*upper secondary	0.332	1.539	0.644	3.68
Colon cancer*compulsory	0.465	1.404	0.565	3.493
Breast cancer*upper secondary	0.576	0.817	0.403	1.658
Breast cancer*compulsory	0.743	0.886	0.429	1.829
Prostate cancer*upper secondary	0.081	0.611	0.351	1.063
Prostate cancer*compulsory	0.23	0.687	0.372	1.269
Diabetes with/without complications*upper secondary	0.312	0.683	0.326	1.43
Diabetes with/without complications*compulsory	0.127	0.554	0.26	1.182
Acute myocardial infarction*upper secondary	0.622	1.16	0.643	2.095
Acute myocardial infarction*compulsory	0.74	0.899	0.48	1.684

Acute cerebrovascular diseases*upper secondary	0.816	0.927	0.491	1.75
Acute cerebrovascular diseases*compulsory	0.671	1.148	0.607	2.173
Congestive heart failure*upper secondary	0.457	0.809	0.463	1.414
Congestive heart failure*compulsory	0.946	1.02	0.578	1.8
COPD or asthma*upper secondary	0.295	1.543	0.686	3.472
COPD or asthma*compulsory	0.642	1.214	0.536	2.75
Osteoarthritis*upper secondary	0.036	0.574	0.343	0.963
Osteoarthritis*compulsory	0.068	0.607	0.356	1.037
Back problems and disc order*upper secondary	0.447	1.225	0.726	2.066
Back problems and disc order*compulsory	0.437	1.234	0.726	2.097
Constant	<.001	0.032		
"-2 Log-Likelihood"	16699.63			
ROC	0.743			
