# Supplementary material for: Social disparities in unplanned 30-day readmission rates after hospital discharge in patients with chronic health conditions: A retrospective cohort study using patient level hospital administrative data linked to the population census in Switzerland
Source: PLoS One. 2022 Sep 22;17(9):e0273342. doi: 10.1371/journal.pone.0273342 (PMC9499293; doi:10.1371/journal.pone.0273342)
Supplement: S2 Table — (PDF) [file pone.0273342.s003.pdf]

S2 Table. Odds ratios of fully adjusted logistic regression for risk of unplanned 30-day readmission (all-condition model C) with interaction chronic condition* household type (N=62,109)

**Outcome: risk for 30-day readmission**	**Model C with CHC*household type**			
	Sig.	OR	95% CI Lower	Upper
Education level				
tertiary (ref.)	0.002			
upper secondary	0.005	1.205	1.057	1.373
compulsory	<.001	1.298	1.122	1.502
Insurance class				
mandatory (ref.)				
(Semi-)private	0.031	0.891	0.802	0.99
Household type				
Living with others (ref.)				
Living alone	0.024	1.443	1.049	1.985
Sex				
Men (ref.)				
Women	0.036	0.894	0.806	0.993
Language skills				
At least regional language or English (ref.)				
Not regional language and no English	0.423	1.058	0.921	1.215
Age				
<= 55 years	<.001			
56-65 years	0.001	1.329	1.125	1.57
67-74 years	<.001	1.612	1.367	1.9
75+ years	<.001	1.988	1.692	2.336
Chronic health condition				
Ischaemic heart disease (ref.)	<.001			
Lung cancer	<.001	6.474	5.007	8.369
Colon cancer	<.001	2.076	1.485	2.901
Breast cancer	0.335	1.169	0.851	1.606
Prostate cancer	<.001	2.111	1.619	2.753
Diabetes with/without complications	0.003	1.739	1.214	2.489
Acute myocardial infarction	<.001	1.62	1.248	2.104
Acute cerebrovascular diseases	<.001	1.765	1.335	2.332
Congestive heart failure	<.001	3.007	2.368	3.82
COPD or asthma	<.001	2.148	1.611	2.866
Osteoarthritis	<.001	0.588	0.463	0.746
Back problems and disc order	0.078	1.228	0.977	1.544
Comorbidity				
NSD, centred by CHC	<.001	1.145	1.108	1.183
Mental comorbidity: no (ref.)				
Mental comorbidity: yes	0.018	1.191	1.03	1.376
Previous hospital stay last 6 months				
No (ref.)				
Yes	<.001	1.777	1.592	1.982
Length of hospital stay (LOS)				
LOS, centred by CHC, Q1 (ref.)	<.001			
LOS, centred by CHC, Q2	0.43	0.945	0.822	1.087
LOS, centred by CHC, Q3	0.253	1.08	0.946	1.233
LOS, centred by CHC, Q4	<.001	1.64	1.448	1.859
CHC*Household type				
Ischaemic heart disease (ref.)	0.651			
Lung cancer*living alone	0.035	0.606	0.381	0.966
Colon cancer*living alone	0.202	0.659	0.348	1.25
Breast cancer*living alone	0.09	0.632	0.372	1.074
Prostate cancer*living alone	0.581	0.861	0.507	1.464
Diabetes with/without complications*living alone	0.982	0.994	0.569	1.736
Acute myocardial infarction*living alone	0.163	0.712	0.442	1.148
Acute cerebrovascular diseases*living alone	0.442	0.83	0.516	1.335
Congestive heart failure*living alone	0.193	0.769	0.517	1.142
COPD or asthma*living alone	0.071	0.647	0.403	1.038
Osteoarthritis*living alone	0.127	0.71	0.456	1.103
Back problems and disc order*living alone	0.108	0.721	0.484	1.074
Constant	<.001	0.011		

Omnibus Chi^2	1556.755(37). <.001
"-2 Log-Likelihood"	16723.28
ROC	0.741
