# Supplementary material for: Social disparities in unplanned 30-day readmission rates after hospital discharge in patients with chronic health conditions: A retrospective cohort study using patient level hospital administrative data linked to the population census in Switzerland
Source: PLoS One. 2022 Sep 22;17(9):e0273342. doi: 10.1371/journal.pone.0273342 (PMC9499293; doi:10.1371/journal.pone.0273342)
Supplement: S3 Table — (PDF) [file pone.0273342.s004.pdf]

S3 Table. Odds ratios of multivariate logistic regression for risk of unplanned 30-day readmission by social factors, health status and length of stay in hospital for all heart diseases and diabetes (N total=19,324°/N readmissions=897)

	A: Social factors				B: Health status				C: Length of stay			
	Sig.	OR	95% CI		Sig.	OR	95% CI		Sig.	OR	95% CI	
			Lower	Upper			Lower	Upper			Lower	Upper
Education level												
tertiary (ref.)	0.005				0.013				0.018			
upper secondary	0.049	1.224	1.001	1.498	0.069	1.207	0.985	1.478	0.069	1.207	0.985	1.478
compulsory	0.001	1.435	1.151	1.789	0.004	1.39	1.114	1.735	0.005	1.374	1.101	1.715
Insurance class												
mandatory (ref.)												
(Semi-)private	0.053	0.854	0.727	1.002	0.095	0.872	0.742	1.024	0.14	0.885	0.753	1.041
Household type												
Living with others (ref.)												
Living alone	0.019	1.198	1.03	1.394	0.027	1.187	1.02	1.382	0.043	1.17	1.005	1.362
Sex												
Men (ref.)												
Women	0.686	0.968	0.829	1.132	0.779	0.978	0.836	1.143	0.735	0.973	0.833	1.138
Age (years)	<.001	1.039	1.033	1.046	<.001	1.033	1.026	1.039	<.001	1.031	1.025	1.038
Comorbidity												
NSD centred by CHC, below average (ref.)*												
average					0.584	0.946	0.775	1.155	0.459	0.927	0.759	1.133
above average					<.001	1.403	1.182	1.666	0.003	1.296	1.089	1.542
Mental comorbidity: no (ref.)												
Mental comorbidity: yes					0.035	1.257	1.016	1.555	0.111	1.19	0.961	1.473
Previous hospital stay last 6 months												
No (ref.)												
Yes					<.001	2.16	1.851	2.521	<.001	2.189	1.875	2.556
LOS, centred by CHC, Q1-Q3 (Ref.)												
LOS, centred by CHC, Q4									<.001	1.643	1.418	1.903
Constant	<.001	0.002			<.001	0.003			<.001	0.003		
Omnibus Chi^2	231.29(6) p<.001				357.16(10) p<.001				399.02(11) p<.001			
"-2 log-likelihood"	7028.08				6902.21				6860.34			
ROC	0.649				0.687				0.697			

°3 missing; *categorized because of insufficient group discrimination
