# Supplementary material for: Social disparities in unplanned 30-day readmission rates after hospital discharge in patients with chronic health conditions: A retrospective cohort study using patient level hospital administrative data linked to the population census in Switzerland
Source: PLoS One. 2022 Sep 22;17(9):e0273342. doi: 10.1371/journal.pone.0273342 (PMC9499293; doi:10.1371/journal.pone.0273342)
Supplement: S4 Table — (PDF) [file pone.0273342.s005.pdf]

S4 Table. Odds ratios of multivariate logistic regression for risk of unplanned 30-day readmission by social factors, health status and length of stay in hospital for ischaemic heart disease (N total=6,872/N readmissions=185)

	A: Social factors				B: Health status				C: Length of stay			
	Sig.	OR	95% CI		Sig.	OR	95% CI		Sig.	OR	95% CI	
			Lower	Upper			Lower	Upper			Lower	Upper
Education level												
tertiary (ref.)	0.413				0.524				0.63			
upper secondary	0.868	0.967	0.648	1.442	0.714	0.927	0.619	1.388	0.795	0.948	0.632	1.421
compulsory	0.387	1.222	0.776	1.923	0.583	1.137	0.718	1.801	0.61	1.128	0.71	1.792
Insurance class												
mandatory (ref.)												
(Semi-) private	0.35	0.857	0.62	1.185	0.631	0.922	0.663	1.283	0.774	0.953	0.683	1.328
Household type												
Living with others (ref.)												
Living alone	0.135	1.293	0.923	1.81	0.215	1.24	0.883	1.74	0.289	1.203	0.855	1.693
Sex												
Men (ref.)												
Women	0.122	1.304	0.931	1.827	0.074	1.364	0.971	1.917	0.082	1.355	0.963	1.908
Age (years)	<.001	1.038	1.022	1.054	<.001	1.03	1.014	1.046	0.001	1.026	1.01	1.042
Comorbidity												
Somatic Comorbidities: 0 (Ref.)					0.04				0.333			
1					0.689	1.096	0.698	1.721	0.813	1.056	0.672	1.661
2					0.048	1.575	1.004	2.469	0.157	1.389	0.882	2.189
3+					0.02	1.719	1.088	2.717	0.176	1.381	0.865	2.207
Mental comorbidity: no (Ref.)												
Mental comorbidity: yes					0.023	1.993	1.098	3.619	0.195	1.496	0.813	2.752
Previous hospital stay last 6 months												
No (Ref.)												
Yes					<.001	3.152	2.285	4.348	<.001	3.18	2.301	4.395
LOS, centred by CHC, Q1-Q3 (Ref.)												
LOS, centred by CHC, Q4									<.001	2.283	1.655	3.149
Constant	<.001	0.002			<.001	0.002			<.001	0.002		
Omnibus Chi^2	43.90(6). <.001				103.65(11). <.001				127.63(12). <.001			
"-2 log-likelihood"	1658.57				1598.82				1574.84			
ROC	0.645				0.706				0.731			
