# Supplementary material for: Social disparities in unplanned 30-day readmission rates after hospital discharge in patients with chronic health conditions: A retrospective cohort study using patient level hospital administrative data linked to the population census in Switzerland
Source: PLoS One. 2022 Sep 22;17(9):e0273342. doi: 10.1371/journal.pone.0273342 (PMC9499293; doi:10.1371/journal.pone.0273342)
Supplement: S5 Table — (PDF) [file pone.0273342.s006.pdf]

S5 Table. Odds ratios of multivariate logistic regression for risk of unplanned 30-day readmission by social factors, health status and length of stay in hospital for congestive heart failure (N total=3,492°/N readmissions=324)

	A: Social factors				B: Health status				C: Length of stay			
	Sig.	OR	95% CI		Sig.	OR	95% CI		Sig.	OR	95% CI	
			Lower	Upper			Lower	Upper			Lower	Upper
Education level												
tertiary (ref.)	0.042				0.048				0.057			
upper secondary	0.118	1.37	0.923	2.034	0.134	1.354	0.911	2.011	0.132	1.357	0.912	2.017
compulsory	0.015	1.667	1.105	2.515	0.018	1.648	1.091	2.488	0.02	1.632	1.08	2.466
Insurance class												
mandatory (ref.)												
(Semi-) private	0.065	1.29	0.985	1.691	0.063	1.293	0.986	1.697	0.043	1.325	1.009	1.739
Household type												
Living with others (ref.)												
Living alone	0.653	1.06	0.823	1.364	0.553	1.08	0.838	1.392	0.604	1.069	0.83	1.379
Sex												
Men (ref.)												
Women	0.316	0.876	0.676	1.135	0.359	0.885	0.682	1.149	0.333	0.879	0.677	1.141
Age (years)												
<74	0.102				0.166				0.173			
74-81	0.059	1.363	0.989	1.878	0.116	1.296	0.938	1.791	0.107	1.305	0.944	1.803
82-87	0.186	1.249	0.898	1.737	0.321	1.183	0.849	1.65	0.349	1.172	0.841	1.635
88+	0.019	1.529	1.073	2.178	0.031	1.477	1.035	2.106	0.036	1.462	1.025	2.087
Comorbidity												
Somatic Comorbidities: 0-2 (ref.)					0.006				0.018			
3					0.332	0.83	0.57	1.209	0.3	0.819	0.562	1.194
4					0.224	1.236	0.879	1.738	0.295	1.2	0.853	1.69
5+					0.018	1.458	1.068	1.989	0.045	1.377	1.007	1.883
Mental comorbidity: no (ref.)												
Mental comorbidity: yes					0.9	1.022	0.73	1.43	0.998	1	0.714	1.402
Previous hospital stay last 6 months												
No (Ref.)												
Yes					0.175	1.19	0.926	1.531	0.187	1.185	0.921	1.525
LOS, centred by CHC, Q1-Q3 (Ref.)												
LOS, centred by CHC, Q4									<.001	1.651	1.279	2.132
Constant	<.001	0.055			<.001	0.047			<.001	0.043		
Omnibus Chi^2	16.02(8) p. <.05				31.31(13) p. <.01				45.35(14) p. <.001			
"-2 log-likelihood"	2141.55				2126.27				2112.23			
ROC	0.563				0.594				0.614			

° 1 Missing: n=2
