# Supplementary material for: Social disparities in unplanned 30-day readmission rates after hospital discharge in patients with chronic health conditions: A retrospective cohort study using patient level hospital administrative data linked to the population census in Switzerland
Source: PLoS One. 2022 Sep 22;17(9):e0273342. doi: 10.1371/journal.pone.0273342 (PMC9499293; doi:10.1371/journal.pone.0273342)
Supplement: S6 Table — (PDF) [file pone.0273342.s007.pdf]

S6 Table. Odds ratios of multivariate logistic regression for risk of unplanned 30-day readmission by social factors, health status and length of stay in hospital for acute myocard infarctus (N total=4,275/N readmissions=166)

	A: Social factors				B: Health status				C: Length of stay			
	Sig.	OR	95% CI		Sig.	OR	95% CI		Sig.	OR	95% CI	
			Lower	Upper			Lower	Upper			Lower	Upper
Education level												
tertiary (ref.)	0.258				0.267				0.294			
upper secondary	0.122	1.413	0.911	2.191	0.136	1.398	0.9	2.17	0.167	1.365	0.878	2.12
compulsory	0.512	1.188	0.71	1.986	0.572	1.16	0.693	1.944	0.669	1.119	0.668	1.876
Insurance class												
mandatory (ref.)												
(Semi-) private	0.197	0.776	0.528	1.141	0.204	0.779	0.53	1.146	0.234	0.791	0.538	1.164
Household type												
Living with others (ref.)												
Living alone	0.921	1.019	0.707	1.468	0.974	0.994	0.689	1.434	0.846	0.964	0.668	1.392
Sex												
Men (ref.)												
Women	0.475	1.145	0.79	1.661	0.389	1.178	0.812	1.71	0.456	1.152	0.794	1.672
Age (years)	<.001	1.04	1.026	1.053	<.001	1.033	1.019	1.047	<.001	1.029	1.015	1.044
Comorbidity												
Somatic Comorbidities: 0 (ref.)					0.013				0.111			
1					0.703	1.095	0.687	1.744	0.826	1.054	0.661	1.681
2					0.697	0.899	0.526	1.536	0.468	0.819	0.477	1.405
3+					0.018	1.773	1.103	2.852	0.14	1.449	0.885	2.371
Mental comorbidity: no (ref.)												
Mental comorbidity: yes					0.216	0.671	0.357	1.261	0.117	0.602	0.319	1.135
Previous hospital stay last 6 months												
no (ref.)												
yes					0.043	1.652	1.015	2.687	0.054	1.614	0.991	2.629
LOS, centred by CHC, Q1-Q3 (ref.)												
LOS, centred by CHC, Q4									0.001	1.819	1.283	2.578
Constant	<.001	0.002			<.001	0.003			<.001	0.004		
Omnibus Chi^2	44.21(6) p<.001				60.81(11) p<.001				71.84(12) p<.001			
"-2 log-likelihood"	1359.78				1343.18				1332.15			
ROC	0.648				0.665				0.681			
