# Supplementary material for: Social disparities in unplanned 30-day readmission rates after hospital discharge in patients with chronic health conditions: A retrospective cohort study using patient level hospital administrative data linked to the population census in Switzerland
Source: PLoS One. 2022 Sep 22;17(9):e0273342. doi: 10.1371/journal.pone.0273342 (PMC9499293; doi:10.1371/journal.pone.0273342)
Supplement: S7 Table — (PDF) [file pone.0273342.s008.pdf]

S7 Table. Odds ratios of multivariate logistic regression for risk of unplanned 30-day readmission by social factors, health status and length of stay in hospital for acute cerebrovascular disease (stroke) (N total=3,109/N readmissions=143)

	**A: Social factors**				**B: Health status**				**C: Length of stay**			
	Sig.	OR	95% CI		Sig.	OR	95% CI		Sig.	OR	95% CI	
			Lower	Upper			Lower	Upper			Lower	Upper
Education level												
tertiary (ref.)	0.143				0.156				0.163			
upper secondary	0.42	1.232	0.742	2.045	0.425	1.233	0.737	2.064	0.421	1.235	0.738	2.068
compulsory	0.07	1.658	0.959	2.869	0.076	1.652	0.948	2.877	0.079	1.645	0.944	2.867
Insurance class												
mandatory (ref.)												
(Semi-)private	0.536	0.879	0.583	1.324	0.578	0.888	0.584	1.35	0.584	0.89	0.585	1.353
Household type												
Living with others (ref.)												
Living alone	0.333	1.206	0.826	1.76	0.443	1.161	0.792	1.702	0.469	1.152	0.786	1.689
Sex												
Men (ref.)												
Women	0.37	0.841	0.575	1.229	0.386	0.844	0.576	1.237	0.358	0.836	0.57	1.226
Age (years)	<.001	1.03	1.015	1.045	0.007	1.021	1.006	1.037	0.01	1.02	1.005	1.036
Comorbidity												
Somatic Comorbidities: 0 (ref.)					0.013				0.015			
1					0.245	1.525	0.749	3.106	0.218	1.567	0.767	3.2
2					0.265	1.495	0.737	3.034	0.247	1.52	0.748	3.089
3+					0.009	2.46	1.257	4.814	0.008	2.477	1.265	4.848
Mental comorbidity: no (ref.)												
Mental comorbidity: yes					0.022	1.672	1.077	2.596	0.027	1.646	1.059	2.559
Previous hospital stay last 6 months												
No (ref.)												
Yes					<.001	3.676	2.48	5.449	<.001	3.698	2.494	5.483
LOS, centred by CHC, Q1-Q3 (Ref.)												
LOS, centred by CHC, Q4									0.399	1.185	0.799	1.758
Constant	<.001	0.005			<.001	0.004			<.001	0.004		
Omnibus Chi^2	28.75(6) p<.001				83.04(11) p<.001				84.74(12) p<.001			
"-2 log-likelihood"	1131.22				1076.93				1076.24			
ROC	0.63				0.72				0.721			
