# Supplementary material for: Social disparities in unplanned 30-day readmission rates after hospital discharge in patients with chronic health conditions: A retrospective cohort study using patient level hospital administrative data linked to the population census in Switzerland
Source: PLoS One. 2022 Sep 22;17(9):e0273342. doi: 10.1371/journal.pone.0273342 (PMC9499293; doi:10.1371/journal.pone.0273342)
Supplement: S8 Table — (PDF) [file pone.0273342.s009.pdf]

S8 Table. Odds ratios of multivariate logistic regression for risk of unplanned 30-day readmission by social factors, health status and length of stay in hospital for all cancer (N total=10,165/N readmissions=516)

	A: Social factors				B: Health status				C: Length of stay			
	Sig.	OR	95% CI		Sig.	OR	95% CI		Sig.	OR	95% CI	
			Lower	Upper			Lower	Upper			Lower	Upper
Education level												
tertiary (ref.)	<.001				0.001				0.001			
upper secondary	0.02	1.35	1.049	1.736	0.038	1.306	1.015	1.682	0.034	1.314	1.02	1.692
compulsory	<.001	1.825	1.375	2.422	<.001	1.709	1.286	2.272	<.001	1.702	1.28	2.263
Insurance class												
mandatory (ref.)												
(Semi-)private	0.077	0.832	0.678	1.02	0.156	0.862	0.702	1.058	0.161	0.863	0.703	1.06
Household type												
Living with others (ref.)												
Living alone	0.194	1.149	0.932	1.416	0.274	1.124	0.911	1.387	0.373	1.101	0.892	1.359
Sex												
Men (Ref.)												
Women	<.001	0.47	0.387	0.571	<.001	0.475	0.391	0.577	<.001	0.466	0.383	0.567
Age (years)	0.005	1.012	1.004	1.021	0.184	1.006	0.997	1.015	0.199	1.006	0.997	1.014
Comorbidity												
NSD, centered by CHC					<.001	1.193	1.111	1.281	<.001	1.155	1.074	1.242
Mental comorbidity: no (ref.)												
Mental comorbidity: yes					0.006	1.576	1.137	2.185	0.011	1.526	1.1	2.117
Previous hospital stay last 6 months												
No (ref.)												
Yes					0.001	1.474	1.17	1.858	0.001	1.467	1.163	1.849
LOS, centred by CHC, Q1-Q3 (Ref.)												
LOS, centred by CHC, Q4									<.001	1.551	1.268	1.896
Constant	<.001	0.025			<.001	0.035			<.001	0.032		
Omnibus Chi^2	111.52(6) p<.001				155.82(9) p<.001				173.27(10) p<.001			
"-2 log-likelihood"	3969.82				3925.51				3908.06			
ROC	0.635				0.663				0.671			
