# Supplementary material for: Social disparities in unplanned 30-day readmission rates after hospital discharge in patients with chronic health conditions: A retrospective cohort study using patient level hospital administrative data linked to the population census in Switzerland
Source: PLoS One. 2022 Sep 22;17(9):e0273342. doi: 10.1371/journal.pone.0273342 (PMC9499293; doi:10.1371/journal.pone.0273342)
Supplement: S9 Table — (PDF) [file pone.0273342.s010.pdf]

S9 Table. Odds ratios of multivariate logistic regression for risk of unplanned 30-day readmission by social factors, health status and length of stay in hospital for lung cancer (N total=1'418/N readmissions=200)

	**A: Social factors**				**B: Health status**				**C: Length of stay**			
	Sig.	OR	95% CI		Sig.	OR	95% CI		Sig.	OR	95% CI	
			Lower	Upper			Lower	Upper			Lower	Upper
Education level												
tertiary (ref.)	0.007				0.01				0.007			
upper secondary	0.008	2.019	1.198	3.401	0.011	1.972	1.168	3.329	0.007	2.07	1.223	3.503
compulsory	0.002	2.418	1.39	4.204	0.002	2.359	1.354	4.109	0.002	2.42	1.387	4.22
Insurance class												
mandatory (ref.)												
(Semi-) private	0.886	1.027	0.712	1.482	0.684	1.079	0.747	1.561	0.681	1.081	0.746	1.565
Household type												
Living with others (ref.)												
Living alone	0.672	0.928	0.658	1.31	0.618	0.915	0.646	1.296	0.542	0.897	0.632	1.272
Sex												
Men (ref.)												
Women	0.154	0.79	0.571	1.093	0.22	0.814	0.587	1.13	0.206	0.809	0.582	1.124
Age (years)	0.522	1.005	0.99	1.02	0.88	0.999	0.983	1.015	0.812	0.998	0.982	1.014
Comorbidity												
Somatic Comorbidities: 0 (ref.)					0.003				0.008			
1					0.026	1.933	1.083	3.451	0.029	1.906	1.067	3.406
2					0.027	1.974	1.081	3.604	0.055	1.81	0.987	3.319
3+					<.001	2.895	1.625	5.159	0.001	2.656	1.485	4.749
Mental comorbidity: no (ref.)												
Mental comorbidity: yes					0.936	0.98	0.589	1.628	0.87	0.958	0.576	1.595
Previous hospital stay last 6 months												
No (ref.)												
Yes					0.401	1.164	0.816	1.659	0.444	1.149	0.805	1.64
LOS, centred by CHC, Q1-Q3 (Ref.)												
LOS, centred by CHC, Q4									0.005	1.636	1.165	2.299
Constant	<.001	0.067			<.001	0.047			<.001	0.045		
Omnibus Chi^2	13.93(6) p<.05				30.03(11) p<.01				37.79(12) p<.001			
"-2 log-likelihood"	1139.91				1123.81				1116.05			
ROC	0.568				0.624				0.635			
