# Supplementary material for: Social disparities in unplanned 30-day readmission rates after hospital discharge in patients with chronic health conditions: A retrospective cohort study using patient level hospital administrative data linked to the population census in Switzerland
Source: PLoS One. 2022 Sep 22;17(9):e0273342. doi: 10.1371/journal.pone.0273342 (PMC9499293; doi:10.1371/journal.pone.0273342)
Supplement: S10 Table — (PDF) [file pone.0273342.s011.pdf]

S10 Table. Odds ratios of multivariate logistic regression for risk of unplanned 30-day readmission by social factors, health status and length of stay in hospital for breast cancer (N total=4'547/N readmissions=101)

	A: Social factors				B: Health status				C: Length of stay			
	Sig.	OR	95% CI Lower	Upper	Sig.	OR	95% CI Lower	Upper	Sig.	OR	95% CI Lower	Upper
Education level												
tertiary (ref.)	0.31				0.537				0.531			
upper secondary	0.576	1.183	0.656	2.136	0.694	1.127	0.622	2.04	0.678	1.134	0.626	2.055
compulsory	0.168	1.577	0.826	3.013	0.318	1.395	0.725	2.684	0.31	1.405	0.729	2.707
Insurance class												
mandatory (ref.)												
(Semi-)private	0.666	0.91	0.592	1.398	0.908	0.975	0.632	1.504	0.909	0.975	0.632	1.505
Household type												
Living with others (ref.)												
Living alone	0.581	1.133	0.728	1.764	0.619	1.119	0.718	1.744	0.743	1.078	0.689	1.685
Age (years)	0.276	1.009	0.992	1.027	0.996	1	0.983	1.018	0.974	1	0.982	1.017
Comorbidity												
Somatic Comorbidities: 0 (ref.)					0.003				0.023			
1					0.108	1.483	0.917	2.398	0.197	1.378	0.846	2.243
2					0.001	2.577	1.439	4.615	0.007	2.281	1.255	4.145
3+					0.004	2.837	1.398	5.757	0.017	2.422	1.17	5.012
Mental comorbidity: no (ref.)												
Mental comorbidity: yes					0.072	1.872	0.945	3.706	0.078	1.849	0.934	3.661
Previous hospital stay last 6 months												
No (ref.)												
Yes					0.178	1.511	0.829	2.754	0.205	1.475	0.809	2.69
LOS, centred by CHC, Q1-Q3 (ref.)												
LOS, centred by CHC, Q4									0.042	1.563	1.015	2.407
Constant	<.001	0.01			<.001	0.012			<.001	0.012		
Omnibus Chi^2	6.77(5) p=.238				26.90(10) p<.01				30.88(11) p=.001			
"-2 log-likelihood"	962				941.88				937.9			
ROC	0.574				0.635				0.637			
