# Supplementary material for: Social disparities in unplanned 30-day readmission rates after hospital discharge in patients with chronic health conditions: A retrospective cohort study using patient level hospital administrative data linked to the population census in Switzerland
Source: PLoS One. 2022 Sep 22;17(9):e0273342. doi: 10.1371/journal.pone.0273342 (PMC9499293; doi:10.1371/journal.pone.0273342)
Supplement: S11 Table — (PDF) [file pone.0273342.s012.pdf]

S11 Table. Odds ratios of multivariate logistic regression for risk of unplanned 30-day readmission by social factors, health status and length of stay in hospital for prostate cancer (N total=2,806/N readmissions=145)

	A: Social factors				B: Health status				C: length of stay			
	Sig.	OR	95% CI		Sig.	OR	95% CI		Sig.	OR	95% CI	
			Lower	Upper			Lower	Upper			Lower	Upper
Education level												
tertiary (ref.)	0.443				0.479				0.515			
upper secondary	0.349	0.827	0.557	1.23	0.29	0.807	0.542	1.201	0.296	0.809	0.544	1.204
compulsory	0.807	1.064	0.645	1.758	0.958	0.986	0.594	1.637	0.893	0.966	0.581	1.605
Insurance class												
mandatory (ref.)												
(Semi-) private	0.402	0.852	0.586	1.239	0.516	0.883	0.606	1.286	0.499	0.878	0.603	1.28
Household type												
Living with others (ref.)												
Living alone	0.296	1.252	0.821	1.909	0.319	1.241	0.812	1.896	0.325	1.237	0.809	1.892
Age (years)	0.149	1.014	0.995	1.033	0.775	1.003	0.983	1.023	0.747	1.003	0.984	1.023
Comorbidity												
Somatic Comorbidities: 0 (ref.)					0.027				0.067			
1					0.182	1.316	0.88	1.968	0.2	1.302	0.87	1.949
2					0.249	1.358	0.807	2.286	0.318	1.305	0.774	2.202
3+					0.003	2.43	1.366	4.322	0.008	2.21	1.231	3.969
Mental comorbidity: no (ref.)												
Mental comorbidity: yes					0.277	1.492	0.725	3.074	0.336	1.427	0.692	2.945
Previous hospital stay last 6 months												
no (ref.)												
yes					0.144	1.394	0.893	2.175	0.17	1.366	0.875	2.135
LOS, centred by CHC, Q1-Q3 (Ref.)												
LOS, centred by CHC, Q4									0.039	1.523	1.022	2.27
Constant	<.001	0.023			<.001	0.038			<.001	0.035		
Omnibus Chi^2	5.84(5) p=.322				19.31(10) p<.05				23.34(11) p<.05			
"-2 log-likelihood"	1135.74				1122.28				1118.24			
ROC	0.558				0.608				0.616			
