# Supplementary material for: Social disparities in unplanned 30-day readmission rates after hospital discharge in patients with chronic health conditions: A retrospective cohort study using patient level hospital administrative data linked to the population census in Switzerland
Source: PLoS One. 2022 Sep 22;17(9):e0273342. doi: 10.1371/journal.pone.0273342 (PMC9499293; doi:10.1371/journal.pone.0273342)
Supplement: S12 Table — (PDF) [file pone.0273342.s013.pdf]

S12 Table. Odds ratios of multivariate logistic regression for risk of unplanned 30-day readmission by social factors, health status and length of stay in hospital for COPD (N total=2115/N readmissions=129)

	A: Social factors				B: Health status				C: Length of stay			
	Sig.	OR	95% CI		Sig.	OR	95% CI		Sig.	OR	95% CI	
			Lower	Upper			Lower	Upper			Lower	Upper
Education level												
tertiary (ref.)	0.081				0.102				0.105			
upper secondary	0.035	2.352	1.062	5.211	0.045	2.265	1.019	5.035	0.047	2.247	1.011	4.996
compulsory	0.127	1.889	0.834	4.279	0.147	1.837	0.808	4.18	0.154	1.818	0.799	4.133
Insurance class												
mandatory (ref.)												
(Semi-) private	0.222	0.747	0.467	1.193	0.181	0.725	0.452	1.162	0.176	0.722	0.45	1.157
Household type												
Living with others (ref.)												
Living alone	0.685	0.922	0.624	1.363	0.541	0.884	0.595	1.312	0.535	0.883	0.595	1.31
Sex												
Men (ref.)												
Women	0.604	0.903	0.614	1.328	0.835	0.959	0.649	1.419	0.825	0.957	0.648	1.414
Age (years)	0.014	1.022	1.004	1.04	0.074	1.017	0.998	1.036	0.09	1.016	0.998	1.035
Comorbidity												
Somatic Comorbidities: 0 (ref.)					0.003				0.005			
1					0.593	0.857	0.487	1.508	0.562	0.846	0.481	1.489
2					0.374	0.758	0.412	1.395	0.348	0.747	0.406	1.374
3+					0.037	1.724	1.034	2.876	0.055	1.656	0.988	2.774
Mental comorbidity: no (ref.)												
Mental comorbidity: yes					0.411	0.808	0.486	1.343	0.379	0.796	0.478	1.324
Previous hospital stay last 6 months												
No (ref.)												
Yes					0.044	1.526	1.012	2.303	0.045	1.524	1.01	2.299
LOS, centred by CHC, Q1-Q3 (Ref.)												
LOS, centred by CHC, Q4									0.222	1.284	0.86	1.918
Constant	<.001	0.008			<.001	0.01			<.001	0.01		
Omnibus Chi^2	13.63(6) p<.05				31.61(11) p<.001				33.06(12) p<.001			
"-2 log-likelihood"	957.97				939.99				938.54			
ROC	0.587				0.645				0.640			
