# Supplementary material for: Social disparities in unplanned 30-day readmission rates after hospital discharge in patients with chronic health conditions: A retrospective cohort study using patient level hospital administrative data linked to the population census in Switzerland
Source: PLoS One. 2022 Sep 22;17(9):e0273342. doi: 10.1371/journal.pone.0273342 (PMC9499293; doi:10.1371/journal.pone.0273342)
Supplement: S13 Table — (PDF) [file pone.0273342.s014.pdf]

S13 Table. Odds ratios of multivariate logistic regression for risk of unplanned 30-day readmission by social factors, health status and length of stay in hospital for back problems (N total=12,691/N readmissions=332)

	A: Social factors				B: Health status				C: Length of stay			
	Sig.	OR	95% CI		Sig.	OR	95% CI		Sig.	OR	95% CI	
			Lower	Upper			Lower	Upper			Lower	Upper
Education level												
tertiary (ref.)	0.02				0.059				0.063			
upper secondary	0.01	1.584	1.118	2.243	0.023	1.501	1.059	2.128	0.023	1.497	1.056	2.123
compulsory	0.007	1.694	1.152	2.491	0.028	1.542	1.047	2.272	0.032	1.527	1.037	2.25
Insurance class												
mandatory (ref.)												
(Semi-) private	0.016	0.724	0.557	0.942	0.03	0.746	0.572	0.972	0.022	0.734	0.563	0.956
Household type												
Living with others (ref.)												
Living alone	0.541	1.082	0.84	1.395	0.877	1.02	0.79	1.318	0.996	0.999	0.773	1.292
Sex												
Men (ref.)												
Women	0.554	0.932	0.737	1.178	0.574	0.935	0.739	1.183	0.494	0.921	0.728	1.166
Age (years)	<.001	1.031	1.023	1.039	<.001	1.02	1.012	1.029	<.001	1.019	1.01	1.028
Comorbidity												
Somatic Comorbidities: 0 (ref.)												
1					0.001	1.616	1.222	2.138	0.001	1.605	1.213	2.123
2					0	1.89	1.355	2.636	<.001	1.839	1.317	2.568
3+					0.001	1.898	1.281	2.813	0.003	1.83	1.234	2.716
Mental comorbidity: no (ref.)												
Mental comorbidity: yes					0.008	1.544	1.118	2.132	0.023	1.46	1.054	2.022
Previous hospital stay last 6 months												
No (ref.)												
Yes					0	1.989	1.495	2.648	<.001	1.961	1.473	2.611
LOS, centred by CHC, Q1-Q3 (Ref.)												
LOS, centred by CHC, Q4									0.011	1.389	1.079	1.788
Constant	<.001	0.003			<.001	0.004			<.001	0.004		
Omnibus Chi^2	88.32(6) p<.001				142.80(11) p<.001				149.08(12) p<.001			
"-2 log-likelihood"	2986.15				2931.68				2925.39			
ROC	0.645				0.684				0.690			
