# Supplementary material for: Social disparities in unplanned 30-day readmission rates after hospital discharge in patients with chronic health conditions: A retrospective cohort study using patient level hospital administrative data linked to the population census in Switzerland
Source: PLoS One. 2022 Sep 22;17(9):e0273342. doi: 10.1371/journal.pone.0273342 (PMC9499293; doi:10.1371/journal.pone.0273342)
Supplement: S14 Table — (PDF) [file pone.0273342.s015.pdf]

S14 Table. Odds ratios of multivariate logistic regression for risk of unplanned 30-day readmission by social factors, health status and length of stay in hospital for osteoarthritis (N total=17,380/N readmissions=229)

	A: Social factors				B: Health status				C: Length of stay			
	Sig.	OR	95% CI		Sig.	OR	95% CI		Sig.	OR	95% CI	
			Lower	Upper			Lower	Upper			Lower	Upper
Education level												
tertiary (ref.)	0.348				0.272				0.268			
upper secondary	0.172	0.789	0.562	1.108	0.112	0.758	0.539	1.066	0.108	0.756	0.538	1.063
compulsory	0.627	0.905	0.605	1.353	0.424	0.848	0.565	1.271	0.395	0.839	0.559	1.258
Insurance class												
mandatory (ref.)												
(Semi-) private	0.396	0.883	0.662	1.177	0.432	0.89	0.667	1.189	0.298	0.857	0.64	1.147
Household type												
Living with others (ref.)												
Living alone	0.647	1.077	0.785	1.477	0.758	1.051	0.765	1.444	0.893	1.022	0.744	1.404
Sex												
Men (ref.)												
Women	0.011	0.692	0.521	0.919	0.015	0.702	0.527	0.934	0.011	0.691	0.519	0.92
Age (years)	<.001	1.05	1.036	1.064	<.001	1.043	1.029	1.057	<.001	1.04	1.025	1.054
Comorbidity												
Somatic Comorbidities: 0 (ref.)					0.022				0.058			
1					0.151	1.263	0.918	1.737	0.179	1.244	0.904	1.712
2					0.007	1.67	1.152	2.42	0.015	1.587	1.093	2.304
3+					0.025	1.749	1.073	2.851	0.055	1.616	0.989	2.641
Mental comorbidity: no (ref.)												
Mental comorbidity: yes					0.11	1.537	0.908	2.601	0.156	1.465	0.864	2.485
Previous hospital stay last 6 months												
No (ref.)												
Yes					<.001	2.437	1.703	3.488	<.001	2.444	1.708	3.499
LOS, centred by CHC, Q1-Q3 (Ref.)												
LOS, centred by CHC, Q4									0.002	1.548	1.17	2.048
Constant	<.001	0.001			<.001	0.001			<.001	0.001		
Omnibus Chi^2	67.22(6) p<.001				102.23(11) p<.001				111.28(12) p<.001			
"-2 log-likelihood"	2370.6				2335.59				2326.53			
ROC	0.647				0.67				0.681			
