# Supplementary material for: Social disparities in unplanned 30-day readmission rates after hospital discharge in patients with chronic health conditions: A retrospective cohort study using patient level hospital administrative data linked to the population census in Switzerland
Source: PLoS One. 2022 Sep 22;17(9):e0273342. doi: 10.1371/journal.pone.0273342 (PMC9499293; doi:10.1371/journal.pone.0273342)
Supplement: S15 Table — (PDF) [file pone.0273342.s016.pdf]

S15 Table. Year of birth of patients with/without unplanned 30-day readmission (N=62,109)

Year of birth	Total sample (n=62,109)		no readmission within 30 days (n=59,990)		readmission within 30 days (n=2,119)	
	n	%	n	%	n	%
<1920	136	0.2	122	0.2	14	0.7
1920-1929	3,694	5.9	3,412	5.7	282	13.3
1930-1939	12,701	20.4	12,066	20.1	635	30
1940-1949	18,381	29.6	17,768	29.6	613	28.9
1950-1959	13,843	22.3	13,499	22.5	344	16.2
1960-1969	8,779	14.1	8,619	14.4	160	7.6
1970-1979	3,475	5.6	3,418	5.7	57	2.7
1980-1989	1,058	1.7	1,044	1.7	14	0.7
1990-1999	42	0.1	42	0.1	0	0
Total	62,109	100	59,990	100	2,119	100
