# Supplementary material for: Social disparities in unplanned 30-day readmission rates after hospital discharge in patients with chronic health conditions: A retrospective cohort study using patient level hospital administrative data linked to the population census in Switzerland
Source: PLoS One. 2022 Sep 22;17(9):e0273342. doi: 10.1371/journal.pone.0273342 (PMC9499293; doi:10.1371/journal.pone.0273342)
Supplement: Attachment — Submitted filename: Response to Reviewers.pdf [file pone.0273342.s017.pdf]

PONE-D-22-02570

Social disparities in unplanned 30-day readmission rates after hospital discharge in patients with chronic health conditions: A retrospective cohort study using patient level hospital administrative data linked to the population census in Switzerland

PLOS ONE

Journal Requirements:

When submitting your revision, we need you to address these additional requirements.

1. Please ensure that your manuscript meets PLOS ONE's style requirements, including those for file naming. The PLOS ONE style templates can be found at https://journals.plos.org/plosone/s/file?id=wjVg/PLOSOne_formatting_sample_main_body.pdf and https://journals.plos.org/plosone/s/file?id=ba62/PLOSOne_formatting_sample_title_authors_affiliations.pdf

Response: *We ensured that our manuscript meets PLOS ONE's style requirements and file naming guidelines.*

2. We note that you have stated that you will provide repository information for your data at acceptance. Should your manuscript be accepted for publication, we will hold it until you provide the relevant accession numbers or DOIs necessary to access your data. If you wish to make changes to your Data Availability statement, please describe these changes in your cover letter and we will update your Data Availability statement to reflect the information you provide.

Response: *The data underlying our results are owned by a third party and we do not have the permission to share the data. We have adapted the data availability statement to conform with the sample text for third-party data, including the necessary contact information that others will need to access the data in the same manner as we did (see Cover Letter). Further, we upload a supporting information file (S1_File) containing the description of the data sources and the specification of the variables that need to be requested from the third-party.*

3. One of the noted authors is a group or consortium SIHOS Team. In addition to naming the author group, please list the individual authors and affiliations within this group in the acknowledgments section of your manuscript. Please also indicate clearly a lead author for this group along with a contact email address.

Response: *We have added this information (see acknowledgements).*

4. Please include captions for your Supporting Information files at the end of your manuscript, and update any in-text citations to match accordingly. Please see our Supporting Information guidelines for more information: http://journals.plos.org/plosone/s/supporting-information.

Response: *We have moved the list of supporting tables from the supporting-information file to the end of the manuscript and completed the list of the captions. We have uploaded separate files for the*

supplementary tables (S1_Table, S2_Table, … S15_Table) and a new S1_File with the supporting information regarding data availability (see 2.).

Additional Editor Comments:

Thank you for submitting your manuscript. The reviewers and I believe it is of potential value for our readers. However, the reviewers have raised a number of very important issues, and their excellent comments will need to be adequately addressed in a revision before the acceptability of your manuscript for publication in the Journal can be determined. We cannot guarantee that your revised paper will be chosen for publication; this would be solely based on how satisfactorily you have addressed the reviewer comments.

Response: *Thank you very much for your time and careful review of our manuscript. The comments helped us to improve the paper.*

[Note: HTML markup is below. Please do not edit.]

Reviewers' comments:

Reviewer's Responses to Questions

Comments to the Author

1. Is the manuscript technically sound, and do the data support the conclusions?

The manuscript must describe a technically sound piece of scientific research with data that supports the conclusions. Experiments must have been conducted rigorously, with appropriate controls, replication, and sample sizes. The conclusions must be drawn appropriately based on the data presented.

Reviewer #1: Yes

Reviewer #2: Yes

---

2. Has the statistical analysis been performed appropriately and rigorously?

Reviewer #1: Yes

Reviewer #2: Yes

---

3. Have the authors made all data underlying the findings in their manuscript fully available?

The PLOS Data policy requires authors to make all data underlying the findings described in their manuscript fully available without restriction, with rare exception (please refer to the Data Availability Statement in the manuscript PDF file). The data should be provided as part of the manuscript or its supporting information, or deposited to a public repository. For example, in addition to summary statistics, the data points behind means, medians and variance measures

should be available. If there are restrictions on publicly sharing data—e.g. participant privacy or use of data from a third party—those must be specified.

Reviewer #1: Yes

Reviewer #2: Yes

---

4. Is the manuscript presented in an intelligible fashion and written in standard English?

PLOS ONE does not copyedit accepted manuscripts, so the language in submitted articles must be clear, correct, and unambiguous. Any typographical or grammatical errors should be corrected at revision, so please note any specific errors here.

Reviewer #1: Yes

Reviewer #2: Yes

---

5. Review Comments to the Author

Please use the space provided to explain your answers to the questions above. You may also include additional comments for the author, including concerns about dual publication, research ethics, or publication ethics. (Please upload your review as an attachment if it exceeds 20,000 characters)

Reviewer #1: The study is an interesting and important contribution to better understand the existing care problem and the inequality that exists in it. In the discussion, it would be great if specific recommendations were given on how re-admission could be prevented. In the abstract, the conclusion that the social differences are mainly explained by lower health literacy or low adherence falls short and neglects the differences caused by material deprivation and higher burdens.

Response: *Thank you very much for these remarks. We have made the recommendations in the conclusion section more explicit. And we changed the conclusion in the abstract as follows: We assume that low socioeconomic status among patients with chronic conditions increases the risk of unplanned 30-day readmission after hospitalisation due to factors related to their social situation (e.g., low health literacy, material deprivation, high social burden), which may negatively affect cooperation with care providers and adherence to recommended therapies as well as hamper active participation in the medical process and the development of a shared understanding of the disease and its cure.*

Reviewer #2: The author's research on social disparities by using Switzerland data is generally well written and I would like to recommend accepting it if the following things can be addressed.

Response: *We are very thankful for your comments on our manuscript and your supportive feedback.*

#1. Social disparities may affect differently between generations. I would like to see birth year distributions to study participants' generations. Also, it would be helpful for generation definitions in Switzerland.

Response: *Thank you for this interesting thought. We added year of birth distribution as supplementary table (S15_Table) and we refer to it in Table 2.*

#2. Statistical guidelines can be cited in the methodology sections. The followings are great candidates:
2-1)
https://scholar.google.com/scholar?hl=ko&as_sdt=0%2C5&q=Methods+for+testing+statistical+differences+between+groups+in+medical+research%3A+statistical+standard+and+guideline+of+Life+Cycle+Committee&btnG=
2-2)
https://scholar.google.com/scholar?hl=ko&as_sdt=0%2C5&q=Regression+analysis+for+continuous+independent+variables+in+medical+research%3A+statistical+standard+and+guideline+of+Life+Cycle+Committee+&btnG=

Response: *Thank you for these interesting publications on statistical guidelines of which we have added the publication on regression analysis (line 164). "We followed recommendations for the reporting of studies using observational routinely-collected health data (RECORD) (43) and guidelines for regression analysis in medical research (44)."*

New citation:
(44) Lee SW. Regression analysis for continuous independent variables in medical research: statistical standard and guideline of Life Cycle Committee. Life Cycle. 2022;2:e3.

#3. One of the main factors of the LOS and readmission is hospitals. How many hospitals are involved and If there are important differences exist between them, I would like to read the reason.

Response: *Thank you for this inspiring remark. We have added the number of hospitals (n=178)(line 305) and the conclusion "This means that there are no large differences in readmission rates between hospitals." to the following section (lines 301-307): "To assess potential clustering of data, we first ran a multilevel logistic regression model to estimate the variance on the second level (hospital level). Given the fact that the intra-class correlation in the null model was .07, dropping to .04 when social variables were added in the model, the clustering was estimated to be below the conventional threshold of .05, indicating substantial evidence of clustering (Heck et al. 2014)."*

6. PLOS authors have the option to publish the peer review history of their article (what does this mean?). If published, this will include your full peer review and any attached files.

If you choose "no", your identity will remain anonymous but your review may still be made public.

Do you want your identity to be public for this peer review? For information about this choice, including consent withdrawal, please see our Privacy Policy.

Reviewer #1: No

Reviewer #2: No

[NOTE: If reviewer comments were submitted as an attachment file, they will be attached to this email and accessible via the submission site. Please log into your account, locate the manuscript record, and check for the action link "View Attachments". If this link does not appear, there are no attachment files.]

While revising your submission, please upload your figure files to the Preflight Analysis and Conversion Engine (PACE) digital diagnostic tool, https://pacev2.apexcovantage.com/. PACE helps ensure that

figures meet PLOS requirements. To use PACE, you must first register as a user. Registration is free. Then, login and navigate to the UPLOAD tab, where you will find detailed instructions on how to use the tool. If you encounter any issues or have any questions when using PACE, please email PLOS at figures@plos.org. Please note that Supporting Information files do not need this step.
